# A Remarkable Woman in Medicine

**Published:** 1896-06

**Authors:** 


### A REMARKABLE WOMAN IN MEDICINE.

DR. GABRIELE BARONIN POSSANNER is the first woman physician to practise medicine in Austria. During a study in Vienna, in the Summer of 1895, we had the pleasure of meeting and becoming acquainted with Dr. Possanner, who is a most charming and accomplished as well as beautiful Austrian woman, of about twenty-seven years of age.

Dr. Possanner's family belongs to the old German nobility and her very careful education was anything but a professional one, as it is not accepted in society that a woman should have any profession whatever. When she first had the idea of studying medicine her family were very much displeased, but, as she remarked, "when they saw that I put all my luck in it, they did not earnestly object."

She prepared for the matriculant examination privately and passed it after two—instead of eight—years' study; then went to Zurich, where she passed the same examination again; then passed the state's examination and took her degree there. While a student in Zurich the Austrian government offered her a scholarship if she would promise to go to Bavaria after finishing, but this she did not accept, because she would only accept if they accepted her as a real Austrian physician. After completing her studies she went to Vienna and was given a position as *operateur* in Professor De Schauta's *Gynäkologie Klinik* for six months, which position was never before held by a woman.

She then entered a petition to the ministry for instruction for admission to the nostrification of her diploma. After six months she received the answer that they could not take her petition into consideration.

In the Spring of 1895 she was offered the position as physician at the *Officers Tochter Institut*, which she did not accept, as she would have no right to treat any but the children at the institute itself, and really the appointment was in effect only that of a school teacher.

In June, 1895, Dr. Possanner entered a petition to parliament, which was very favorably accepted and given to the government with a warm recommendation. She then entered a petition to the emperor for admission to practise. The emperor sent it with "the great signature," the best recommendation he can give, to the government, and they wanted then to give her a limited admission to practise in women's diseases, without examination. To this she objected, because it would have left the question unsolved for women altogether, and she felt that it did not correspond with the dignity of a doctor to be limited.

Therefore, they had to unroll the principal question of women being admitted to the examination, which is now decided in the best sense possible, as the law passed on March 31, 1896, and Dr. Possanner has before this date passed the required examination for women physicians to practise in Austria.

